# Application of High-Resolution Flat Detector Computed Tomography in Stent Implantation for Intracranial Atherosclerotic Stenosis

**DOI:** 10.3389/fnins.2021.655594

**Published:** 2021-08-27

**Authors:** Tengfei Li, Yuting Wang, Ji Ma, Michael Levitt, Mahmud Mossa-Basha, Chengcheng Shi, Yuncai Ran, Jianzhuang Ren, Xinwei Han, Chengcheng Zhu

**Affiliations:** ^1^Department of Interventional Radiology, First Affiliated Hospital of Zhengzhou University, Zhengzhou, China; ^2^Interventional Institute of Zhengzhou University, Zhengzhou, China; ^3^Department of Radiology, Sichuan Provincial People’s Hospital, University of Electronic Science and Technology of China, Chengdu, China; ^4^Department of Neurological Surgery, University of Washington, Seattle, WA, United States; ^5^Department of Radiology, University of Washington, Seattle, WA, United States; ^6^Department of Magnetic Resonance, First Affiliated Hospital of Zhengzhou University, Zhengzhou, China

**Keywords:** flat detector CT, intracranial atherosclerosis, stenosis, stent, complications

## Abstract

**Objective:**

To evaluate the utility of high-resolution flat-detector computed tomography (HR-FDCT) compared with conventional flat-detector computed tomography (FDCT) for stent placement in symptomatic intracranial atherosclerotic stenosis (ICAS).

**Methods:**

We retrospectively reviewed the clinical data of 116 patients with symptomatic ICAS who underwent stent implantation. Images were acquired using conventional FDCT [voxel size = 0.43 mm (isotropic)] and HR-FDCT [voxel size = 0.15 mm (isotropic)]. Immediately after stent deployment, dual-volume three-dimensional (3D) fusion images were obtained from 3D digital subtraction angiography (DSA) and HR-FDCT. The image quality for stent visualization was graded from 0 to 2 (0: not able to assess; 1: limited, but able to assess; 2: clear visualization), and the stent-expansion status (“full,” “under-expanded” or “poor apposition”) was recorded.

**Results:**

A total of 116 patients with symptomatic ICAS were treated successfully using 116 stents (58 Neuroform^TM^ EZ, 42 Enterprise^TM^, and 16 Apollo^TM^). The mean pre-stent stenosis was 80.5 ± 6.4%, which improved to 20.8 ± 6.9% after stenting. Compared with FDCT, HR-FDCT improved visualization of the fine structures of the stent to improve the image quality that significantly (mean score: 1.63 ± 0.60 *vs*. 0.41 ± 0.59, *P* < 0.001). In 19 patients, stent under-expansion (*n* = 11) or poor apposition (*n* = 8) was identified by HR-FDCT but not by conventional FDCT. After balloon dilatation, stent malapposition was shown to have improved on HR-FDCT. None of the 19 patients with stent malapposition experienced short-term complications during hospitalization or had in-stent stenosis at 6-month follow-up.

**Conclusion:**

High-resolution flat-detector computed tomography (HR-FDCT) improves visualization of the fine structures of intracranial stents deployed for symptomatic ICAS compared with that visualized using conventional FDCT. High-resolution flat-detector computed tomography improves assessment of stent deployment and could reduce the risk of complications.

## Introduction

Intracranial atherosclerotic stenosis (ICAS) is a major cause of ischemic stroke. In the USA, ICAS accounts for 5–10% of ischemic strokes, and it is a more common stroke etiology in Asian, African, and Hispanic populations (Gorelick et al., 2008). Intracranial atherosclerotic stenosis is the source of stroke in as many as 30–50% of cases among Chinese populations (Leung et al., 1993).

Initially, ICAS is treated with non-invasive medical therapy that seeks to reduce the risk of cerebral infarction (Qureshi and Caplan, 2014; Heit and Wintermark, 2018). However, patients who develop recurrent cerebral infarction or recurrent transient ischemic attacks (TIAs) despite optimal medical therapy may be considered for more aggressive treatment, including cerebral arterial angioplasty and/or stent placement (Qureshi and Caplan, 2014; Heit and Wintermark, 2018). Optimal visibility of the intracranial stent is limited using digital subtraction angiography (DSA) applications (Kato et al., 2020). Although some radiopaque elements of intracranial stents may be observed *via* fluoroscopy during treatment, local distortion or incomplete expansion of the stent may be difficult to identify due to limited angiographic angles or the presence of bony or metal obstructions (Li et al., 2019). Incomplete expansion of the stent is an important potential risk factor for stent thrombosis and prolonged duration of stent endothelialization (Foin et al., 2014). Improving intraprocedural imaging of stent placement during ICAS treatment could minimize risks and improve outcomes (Zaidat et al., 2015; Wang and Wang, 2017; Alexander et al., 2019).

High-resolution flat-detector computed tomography (HR-FDCT) technology (syngo DynaCT Micro, Siemens Healthineers, Forchheim, Germany) was developed recently. This technology allows for improved visualization of fine anatomical features or the features of implanted devices during angiographic procedures. Compared with conventional FDCT, HR-FDCT provides higher spatial resolution and reduced artifacts from the cone beam (Yuki et al., 2016; Li et al., 2019). Instead of 2 × 2-pixel binning during read-out from flat-detector imaging, this technology employs a non-binning technique. The exposure conditions and image-processing algorithms have been optimized, and the spatial resolution improved from 0.43 to 0.15 mm.

High-resolution flat-detector computed tomography (HR-FDCT) has been reported to improve the visibility of various intracranial stents for stent-assisted aneurysm embolization when compared with that obtained with conventional FDCT (Yuki et al., 2016; Shintai et al., 2018; Li et al., 2019; Zhang et al., 2019). However, *in vivo* research on the use of this technique to evaluate stent placement for symptomatic ICAS is scarce.

We assessed the *in vivo* image quality of HR-FDCT, its utility for guiding intravascular procedures, and the possible influence on the postoperative outcome. In this way, we aimed to evaluate comprehensively the utility of HR-FDCT for stent placement in symptomatic ICAS.

## Materials and Methods

### Ethical Approval of the Study Protocol

The protocol for this retrospective study was approved by the Ethics Committee of the Biomedical Research Department within First Affiliated Hospital of Zhengzhou University (Zhengzhou, China). The procedures followed were in accordance with the Helsinki Declaration of 1975 and its later amendments.

### Inclusion and Exclusion Criteria

Patients were eligible for inclusion in this study if: (i) a TIA or minor stroke (Fischer et al., 2010) had occurred <90 days before stent placement and was attributed to an angiographically verified >50% stenosis of a major intracranial artery supplying the stroke territory; (ii) the stenosis was located in the intracranial internal carotid artery, M1 segment of the middle cerebral artery, intracranial vertebral artery, or basilar artery, and the diameter of the artery adjacent to the stenosis was 2.0–4.0 mm; (iii) the National Institutes of Health Stroke Scale (NIHSS) score was <9 upon presentation; (iv) patient age was 30–80 years; (v) the patient had at least one risk factor for atherosclerosis (hypertension, diabetes mellitus, hyperlipidemia, hyperhomocysteinemia, or smoking); (vi) ischemic stroke or TIA occurred despite the patient receiving standard medical therapy (antiplatelet or anticoagulant medication); (vii) symptomatic ICAS was treated with a single intracranial stent: Neuroform EZ^TM^ (Stryker Neurovascular, Fremont, CA, United States), Enterprise^TM^ (Codman Neuro, Raynham, MA, United States), or Apollo^TM^ (MicroPort Medical, Shanghai, China).

Patients were excluded if they: (i) were treated with a single or combined drug-eluting balloon or drug-eluting stents; (ii) underwent stenting combined with bypass surgery; (iii) had clinically defined non-atherosclerotic ICAS (e.g., vasculitis, arterial dissection).

### Patient Selection

Consecutive patients who underwent stent implantation in First Affiliated Hospital of Zhengzhou University between June 2017 and December 2018 for treatment of symptomatic ICAS were identified retrospectively by reviewing medical records.

Demographic (age, sex) and clinical information, (risk factors for atherosclerosis) were recorded. Lesion characteristics, including the location and Mori classification, were documented. With regard to the Mori classification, type-A lesions were concentric and <5 mm in length, type-B lesions were eccentric lesions 5–10 mm in length, and type-C lesions were longer than 10 mm and excessively tortuous (Mori et al., 1998).

### Image Acquisition

Preprocedural preparation and the stenting procedure were undertaken as described previously (Jiang et al., 2007; Vajda et al., 2012; Xu et al., 2019), and included clopidogrel (p.o.) for 3-6 months and long-term aspirin (p.o.). Preprocedural vascular stenosis and postoperative residual stenosis were measured manually using calipers on images from catheter angiography.

Immediately after stent deployment, FDCT and HR-FDCT were carried out using the same imaging unit as a routine procedure in our institution for optimal care of patients. Whole-brain FDCT was acquired to exclude parenchymal or subarachnoid hemorrhage, and to determine whether to add heparin and/or tirofiban into postprocedural treatment. We used the following parameters for whole-brain FDCT: acquisition time = 20 s; X-ray tube voltage = 109 kV; tube current = 460 mA; total angle = 200°; 496 frames; zoom size = 48 cm; voxel size = 0.49 mm (isotropic).

Subsequently, HR-FDCT was acquired with the region of interest limited to the stent-placement area (to verify that the stent was fully expanded) using the following parameters: acquisition time = 20 s; X-ray tube voltage = 109 kV; tube current = 460 mA; total angle = 200°; 496 frames; zoom size = 22 cm; voxel size = 0.15 mm (isotropic). The radiation dose for FDCT was 85.3 mGy, and that for HR-FDCT was 294 mGy.

Three-dimensional FDCT and HR-FDCT images were reconstructed automatically on a three-dimensional (3D) workstation (Siemens Healthineers).

After the two acquisitions stated above had been completed, patients underwent 3D-DSA for 5 s for confirmation of vessel patency. 3D-DSA was done using the following parameters: acquisition time for each rotation = 5 s; X-ray tube voltage = 70 kVp; tube current = 460 mA, total angle = 200°; 397 frames; zoom size = 48 cm; voxel size = 0.43 mm (isotropic). For contrast-enhanced DSA, undiluted contrast media (370 mg/mL; Ultravist^TM^; Bayer, Bayer Leverkusen, Germany) was injected at 2.5 mL/s for 5 s, and the scan was started with a 1-s delay. 3D-DSA images and HR-FDCT were fused and displayed as dual-volume (syngo 3D–3D fusion, Siemens Healthineers) to evaluate the vessel-wall apposition of the implanted stent. During 3D–3D fusion, the 3D-DSA images were first reconstructed into two volumes (i.e., we subtracted the volume of 3D vessels and the mask volume of the skull and brain tissues) with identical 3D coordinates. Then, the mask volume was fused with HR-FDCT based on rigid registration of bony structures. Hence, a fusion matrix between 3D-DSA and HR-FDCT was generated. Through application of the same fusion matrix between the subtracted volume and HR-FDCT, the 3D-vessel could be fused with a clearly reconstructed stent from HR-FDCT. If malapposition of the stent was observed, the interventionalist carried out balloon angioplasty to expand the stent further. Follow-up HR-FDCT could be undertaken to confirm stent deployment after angioplasty depending on the interventionalist’s clinical judgment. Stent malapposition was described as “stent under-expansion” (partially opened stent) or “poor apposition” (presence of a gap between the stent trunk and the parent-artery wall, or a gap between the distal or proximal flare of the stent and the parent artery).

### Stenosis Evaluation

The preoperative and postoperative/residual stenosis was calculated according to the warfarin–aspirin symptomatic intracranial disease (WASID) method (Samuels et al., 2000) on DSA images, The success of stenting was defined as complete coverage of the lesion by the stent, resulting in a residual stenosis of ≤30% with good anterograde blood flow.

### Retrospective Evaluation of the Image Quality of FDCT and HR-FDCT

Patients were divided into three groups based on the type of stent deployed (Neuroform, Enterprise, or Apollo). For each stent group, two sets of 3D images (FDCT and HR-FDCT) were anonymized and randomized for evaluation after all procedures had been completed for the included patients. Two experienced neuro-interventionalists (with 12 years and 36 years of experience, respectively) blinded to the procedural results evaluated the image quality independently.

**FIGURE 1:**
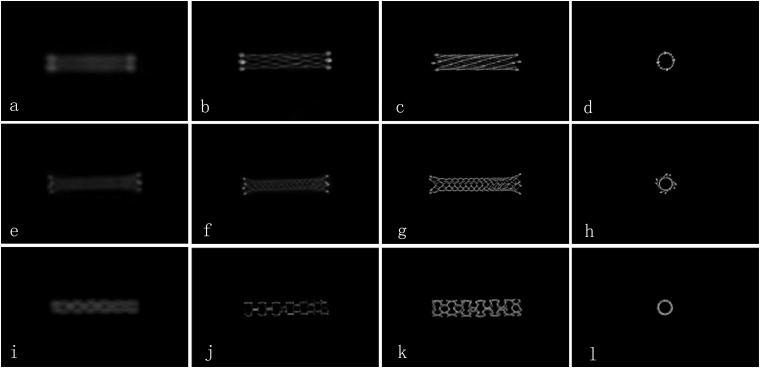
Comparison of the image quality for different types of stents by FDCT and HR-FDCT *in vitro* (representative cases). **(a–d)** Neuroform^TM^ EZ stent with image-quality scores of 0, 1, 2, and 2 points; **(e–h)** Enterprise^TM^ stent with image-quality scores of 0, 1, 2, and 2 points; **(i–l)** Apollo^TM^ stent with image-quality scores of 0, 1, 2, and 2 points. Panels **(a,e,i)** were images by FDCT, and the rest were images by HR-FDCT.

The evaluation criteria were: 0 (the image quality was poor and defined as inadequate delineation between the stent lumen and stent strut, or no visualization of the stent lumen); 1 (the image quality was good and defined as visualization of a mildly inhomogeneous stent lumen, but with the patency of the stent lumen interpretable); 2 (images of excellent quality, the lesion area appeared as a homogeneous stent lumen with clear delineation of the stent strut) (Figure 1) (Jang et al., 2012).

### Postoperative Management and Follow-Up

Vital signs were monitored after stenting. Systolic pressure was maintained at 90–110 mmHg for >48 h. If the NIHSS score or modified Rankin Scale (mRS) score changed, CT and/or magnetic resonance imaging of the head was done. At 1-month clinical follow-up, the scores for the mRS and NIHSS were used to assess functional outcome.

### Statistical Analyses

SPSS 22.0 (IBM, Armonk, NY, United States) was used for statistical analyses. Data with a normal distribution and continuous data are presented as the mean ± SD. Ratings for image quality were evaluated using chi-square or Fisher’s exact tests. Inter-observer agreement between the two raters was analyzed using Kappa (≥ 0.75 was considered “good agreement”) values. *P* < 0.05 was considered significant.

## Results

### Patients and Stenting

Consecutive ICAS patients (*n* = 146) were screened for inclusion. Thirty patients were excluded from the study [12 patients with deployment of single or combined drug-eluting balloons or drug-eluting stents; 18 patients without follow-up DSA/computed tomography angiography (CTA)]. Hence, 116 patients (71 males and 45 females; mean age, 54 ± 11 years) formed the study cohort. The median time from the initial TIA or stroke event to stent placement was 21.7 (range, 14–56) days. The characteristics of patients at baseline are shown in Table 1.

**TABLE 1:** Baseline patient characteristics (*n* = 116).

Baseline characteristics	No. (%)
Age	54 ± 11
Male	71 (61.2)
Female	45 (38.8)
**Risk factor**	
Hyperlipidemia	87(75.0)
Hypertension	77(66.4)
Smoking	52 (44.8)
Diabetes mellitus	31 (26.7)
Hyper-homocysteinemia	29 (25.0)
**Other factors**	
Qualifying stroke event	47 (40.5)
Relevant regional infarct	89 (76.7)
**Lesion location**	
Internal carotid artery	22 (19.0)
Middle cerebral artery	36 (30.0)
Vertebral artery	26 (22.4)
Basilar artery	32 (25.6)
**Lesion morphology**	
Mori Type A	49 (42.2)
Mori Type B	56 (48.3)
Mori Type C	11 (9.5)

Overall, 116 patients were treated with 116 intracranial stents (58 Neuroform EZ, 42 Enterprise, and 16 Apollo). Mean preprocedural stenosis was 80.5 ± 6.4%, and mean postprocedural residual stenosis was 20.8 ± 6.9% (internal carotid artery 19.1 ± 6.8%, middle cerebral artery 22.3 ± 7.6%, vertebral artery 20.4 ± 6.7%, basilar artery 18.9 ± 5.2%). The success rate for the technical procedure was 100%.

### Comparison Between FDCT and HR-FDCT

**FIGURE 2:**
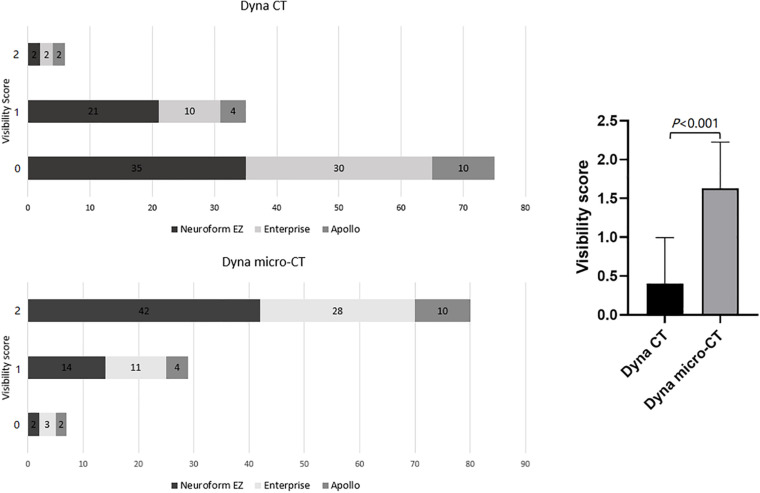
Comparison of the stent visibility scores using FDCT and HR-FDCT (Neuroform EZ stent, *n* = 58; Enterprise stent, *n* = 42; Apollo stent, *n* = 16).

**FIGURE 3:**
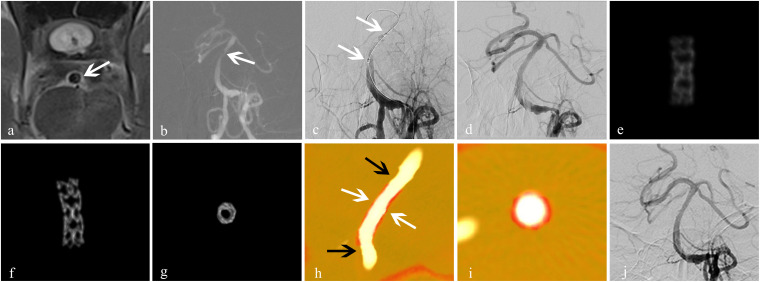
Treatment for an atherosclerotic stenosis in the basilar artery with the Apollo^TM^ stent. A 56-year-old man presented with dizziness of 2-week duration. **(a)** High-resolution MRI showed stenosis of the basal-artery trunk (white arrow); **(b)** 2D-DSA showed severe stenosis (80%) of the basilar-artery trunk (white arrow); **(c)** an Apollo balloon-expandable stent measuring 2.5 mm × 8 mm was placed (white arrow); **(d)** DSA showed improved blood flow with good expansion of the stent, and residual stenosis was 5%; **(e)** conventional FDCT showed the blurred metal struts of the stent with an image-quality score of 0; **(f,g)** HR-FDCT showed that the stent struts were clear and expanded completely, and image quality was improved significantly with a score of 2; **(h,i)** Dual volume three-dimensional fusion images showed good apposition of the stent to the vessel wall (stent, white arrow; vessel wall, black arrow); **(j)** follow-up DSA at 6 months showed durable stent patency. Panels **(e–g)** were obtained *in vivo*.

**FIGURE 4:**
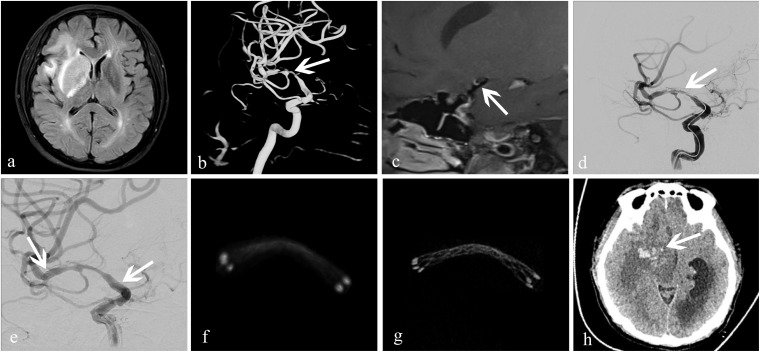
Treatment of an atherosclerotic stenosis in the right middle cerebral artery with the Enterprise^TM^ stent. A 52-year-old woman who presented with left-limb weakness of 12-day duration. **(a)** MRI showed subacute cerebral infarction in the right temporal lobe and basal ganglia; **(b)** 3D-DSA revealed severe stenosis (83%) at the bifurcation of the right middle cerebral artery (white arrow); **(c)** high-resolution MRI showed that the stenosis was atherosclerotic (white arrow); **(d,e)** dilation using a Gateway^TM^ balloon was done followed by insertion of an Enterprise stent measuring 4.5 mm × 22 mm (white arrow). Residual stenosis was 28%; the two arrows in **(e)** showed both ends of the stent. **(f)** conventional FDCT showed a good position of the distal and proximal stent markers, but the stent itself was poorly visualized with an image-quality score of 0; **(g)** HR-FDCT showed good deployment of the stent, and the image quality was improved significantly with a score of 2; **(h)** 6-h later, CT of the head showed cerebral parenchymal hemorrhage (white arrow) in the former infarction area at right basal ganglia.

**FIGURE 5:**
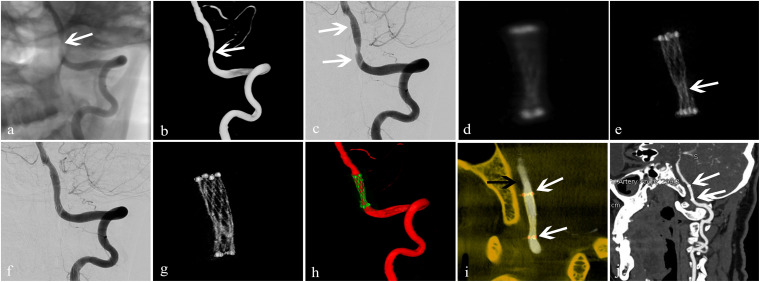
Treatment of an arteriosclerotic stenosis of the V4 segment of the left vertebral artery using the Neuroform^TM^ EZ stent. A 67-year-old man presented with dizziness and instability while standing of 6-day duration. **(a,b)** 2D and 3D-DSA showed 78% stenosis of the V4 segment of the left vertebral artery (white arrow); **(c)** dilation using a Gateway^TM^ balloon was done followed by insertion of a Neuroform EZ stent measuring 3.0 mm × 15 mm (white arrow) with 50% residual stenosis; **(d)** conventional FDCT showed full expansion of the proximal and distal stent markers but poor visualization of the stent itself with an image-quality score of 0; **(e)** HD-FDCT showed the stent was partially expanded with an image-quality score of 1 (white arrow); **(f)** angioplasty with a Gateway balloon was undertaken again with a 3.0 mm × 9 mm balloon to treat stent under-expansion. Review by 2D-DSA showed that stenosis was improved significantly (residual stenosis = 11%); **(g)** after balloon angioplasty, HD-FDCT was done again and confirmed that the stent was fully expanded; **(h,i)** Dual-volume 3D fusion images showed good apposition of the stent to the vessel wall (stent, white arrow; vessel wall, black arrow); **(j)** 6-month follow-up CTA showed durable stent patency (white arrow).

Both readers showed excellent agreement for assessment of image quality (κ = 0.92). The inter-rater agreement was excellent for each type of stent used (κ = 0.89 for Neuroform EZ, 0.94 for Enterprise, and 0.95 for Apollo). Compared with reconstructed images from FDCT, reconstructed images from HR-FDCT showed significant improvement in visualization of stent structure and assessment of stent deployment (mean score: 1.63 ± 0.60 *vs*. 0.41 ± 0.59, *P* < 0.001) (Table 2 and Figures 2–5). The improvement of image quality was significant (*P* < 0.05) for each stent type.

**TABLE 2:** Evaluation of images obtained with FDCT and HR-FDCT (Neuroform EZ stent, *n* = 58; Enterprise stent, *n* = 42; Apollo stent, *n* = 16).

Stent	Visibility score^a^	Method		*P*	Average Score of Image quality	
		Dyna CT	Dyna micro-CT		Dyna CT	Dyna micro-CT
Total	0	75	7	< 0.001*	0.41 ± 0.59	1.63 ± 0.60
	1	35	29			
	2	6	80			
Neuroform EZ	0	35	2	< 0.001*	0.43 ± 0.57	1.69 ± 0.54
	1	21	14			
	2	2	42			
Enterprise	0	30	3	< 0.001*	0.33 ± 0.57	1.60 ± 0.63
	1	10	11			
	2	2	28			
Apollo	0	10	2	0.005*	0.50 ± 0.73	1.5 ± 0.73
	1	4	4			
	2	2	10			

*^∗^*p* < 0.05, Significant difference in quality between FDCT and HR-FDCT images (Neuroform EZ stent, *p* < 0.001; Enterprise stent, *p* < 0.001; Apollo stent, *p* = 0.005). ^*a*^0 = the image quality was poor and defined as inadequate delineation between the stent lumen and stent strut due to severe beaming artifacts, as well as no visualization of the stent lumen due to image noise; 1 = the image quality was good and defined as visualization of a mildly inhomogeneous stent lumen, but with the patency of the stent lumen interpretable; 2 = images of excellent quality, the lesion area appeared as a homogeneous stent lumen with clear delineation of the stent strut.*

### Balloon Angioplasty

By reviewing the HR-FDCT images during the interventional procedure, patients who had stent malapposition were identified and underwent additional balloon angioplasty. According to the reconstruction images obtained from HR-FDCT, 11 stents (seven Neuroform EZ and four Enterprise) were found to be under-expanded. The involved arterial segments were four in intracranial carotid arteries, three in middle cerebral arteries, one in the basilar artery, and three in vertebral arteries. After balloon angioplasty, all stents expanded fully (Figure 5). According to the reconstruction of 3D fusion images obtained from 3D-DSA combined with HR-FDCT, eight stents (four Neuroform EZ and four Enterprise) were found to have poor apposition. The involved arterial segments were four in intracranial carotid arteries, two in middle cerebral arteries, and two in vertebral arteries. After balloon angioplasty, stent malapposition improved, as confirmed by an additional HR-FDCT scan.

### Complications During Hospitalization

One patient had postprocedural intraparenchymal hemorrhage and was discharged 50 days later with a moderate permanent neurological deficit (NIHSS score = 6, mRS score = 3). Six patients had postprocedural ischemic stroke, though DSA confirmed patency in blood flow through the stents in all cases and stroke was assumed to be a result of perforator occlusion based on the infarct pattern. Of these six patients, three had a permanent minor neurological deficit with a mRS score of 1, 1, and 2 and NHISS score of 1, 1, and 2, respectively, at 1 month. There were no cases of acute thrombosis or hemorrhagic complications in the remaining 109 patients. There were no complications among the 19 patients that necessitated balloon angioplasty.

### Follow-Up

A total of 114 patients had adequate follow-up data after surgery. The duration of follow-up was 6–25.3 (median, 12.9) months. The mRS score at 6 months was 0 (*n* = 88), 1 (*n* = 24), 2 (*n* = 1), and 3 (*n* = 1). In the follow-up window, 3.5% (4/114) of patients had ischemic strokes, of which one resulted in permanent disability and three were minor strokes. Also, 3.5% (4/114) of patients had a TIA. Mortality was 2.6% (3/114), none of which were from neurological causes.

A total of 97 patients had follow-up imaging data at 6 months (36 cases by CTA, 61 by DSA); 14 (14.4%) (seven Neuroform EZ, five Enterprise, and two Apollo) showed in-stent stenosis, of which six were symptomatic; three of those were treated with balloon angioplasty. The remaining eight asymptomatic cases were treated conservatively. Regarding the causes of in-stent stenosis, three patients had poor control of hyperglycemia or hypertension, and one patient had poor medication compliance. In the remaining 10 patients, the possible causes were a relatively higher degree of post-stent residual stenosis, vascular endothelial injury due to balloon angioplasty, poor response to antiplatelet medication, and/or prolonged time of vascular endothelialization.

In the 19 patients with stent malapposition who underwent balloon angioplasty, a deterioration of neurological function was not observed in the clinical follow-up (mean, 9.1 months). In addition, in-stent stenosis was not observed at 6-month follow-up DSA.

## Discussion

HR-FDCT has helped improve stent visualization during neurointerventional procedures (Clarençon et al., 2017; Kuriyama et al., 2018; Srinivasan et al., 2018). Studies have explored its utility in patients with intracranial aneurysms [(Caroff et al., 2014; Shintai et al., 2018; Kato et al., 2020). However, few reports have focused on the clinical application of HR-FDCT in stent placement for symptomatic ICAS.

In our relatively large patient cohort (*n* = 116), HR-FDCT improved the visualization of fine structures of the stent, which helped detection of stent malapposition. Nineteen cases of stent under-expansion or poor apposition were identified by HR-FDCT but were not seen by FDCT because of artifacts caused by the metal in the wire mesh and lower spatial resolution. After balloon angioplasty, stent malapposition improved on HR-FDCT. None of the 19 patients with stent malapposition experienced short-term complications during hospitalization or had in-stent stenosis at 6-month follow-up.

Stent under-expansion and poor apposition can lead to an increased risk of acute thrombosis or delayed ischemia after deployment. Balloon angioplasty is often necessary if malapposition is detected (Sheng et al., 2014). Flat-detector computed tomography and HR-FDCT could be utilized for different purposes according to their strengths and limitations. Flat-detector computed tomography could be employed to visualize the whole brain for exclusion of intracranial bleeding due to its large field of view (can be up to 30 × 40 cm) but has limited spatial resolution when compared with HR-FDCT. The latter could be used only for visualizing a small field of view (16 × 16 cm) to compromise for signal read-out burden if pixel binning was not adopted. Caroff and colleagues used HR-FDCT (Vaso CT; Philips, Amsterdam, the Netherlands) to observe stent-assisted embolization of intracranial aneurysms. They found that HR-FDCT could be used to assess stent deployment and reduce the risk of thromboembolic events (Caroff et al., 2014). We garnered similar findings in that HR-FDCT aided stent apposition, and serious complications (e.g., acute thrombosis) did not occur during or after intervention.

Among the six patients in our study with ischemic stroke during the perioperative period, three had basilar-artery stenosis and the post-stenting infarctions were located in the pons. The remaining three cases had a stenosis in the middle cerebral artery and the post-stenting infarctions were located in the basal ganglia. Considering that (i) postprocedural DSA did not show an apparent filling defect in the stent or large-artery occlusion, and (ii) the infarct pattern, perforator territory infarcts involving pontine and medial lenticulostriate perforators were favored.

The Stenting vs. Aggressive Medical Management for Preventing Recurrent Stroke in Intracranial Stenosis (SAMMPRIS) trial reported a high prevalence of periprocedural complications in the stenting arm (14.7% higher than that in the medical-therapy arm) (Derdeyn et al., 2014). However, the Post Market Surveillance Study of the Wingspan Stent System (WEAVE) trial reported a much lower prevalence of periprocedural complication (2.6%) for on-label intracranial stenting. That result suggested that the previously reported high prevalence of complications might have been due to inexperienced interventionalists, poor selection of patients, and immature standards of practice for intracranial stenting (Alexander et al., 2019). Those results indicated that careful selection of patients and experienced operators, if aided by additional tools such as HR-FDCT, can achieve excellent safety and efficacy for ICAS stenting.

Post-stent restenosis is an important factor affecting the extension and application of stenting in ICAS. We found that the overall prevalence of stent restenosis and prevalence of restenosis of a single stent type were lower than those reported by a multicenter, large-sample study which used FDCT (Jiang et al., 2012). In that study, the overall prevalence of stent restenosis was 23.3%, whereas that for the Apollo stent was 20.3% and that for the self-expandable stent was 27.9%. Many variables were involved in the two studies, but the lower prevalence of restenosis in our study (14.4% overall) could be attributed (at least in part) to the use of HR-FDCT as well as the strict criteria on which stent placement was decided.

Our study had three main limitations. First, this was a single-center, retrospective study, and some selection biases may have been present. Second, the prevalence of restenosis may have been over- or underestimated because only 53.5% (61/114) of patients underwent follow-up studies using angiography. Third, the Wingspan stent-delivery system was not used in the present study. Although it is approved for intracranial stenosis in the USA, it has been criticized for its rigidity and open-cell design with radial force (Feng et al., 2015; Du et al., 2018). Off-label use of several other types of intracranial stents has been used to treat intracranial stenosis [e.g., Neuroform (Du et al., 2018; Xu et al., 2019) and Enterprise (Feng et al., 2015; Huang et al., 2019; Salik et al., 2019)].

## Conclusion

High-resolution flat-detector computed tomography (HR-FDCT) improves visualization of the fine structures of intracranial stents deployed for symptomatic ICAS compared with that using conventional FDCT. High-resolution flat-detector computed tomography improves assessment of stent deployment and could reduce the risk of complications.

## Data Availability Statement

The original contributions presented in the study are included in the article/supplementary material, further inquiries can be directed to the corresponding authors.

## Ethics Statement

The studies involving human participants were reviewed and approved by Ethics committee of Biomedical Research of the First Affiliated Hospital of Zhengzhou University. The patients/participants provided their written informed consent to participate in this study.

## Author Contributions

TL, JM, CS, YR, and JR were responsible for patient recruitment, data acquisition, and/or analysis. TL, YW, XH, and CZ contributed to the study design. TL contributed to the imaging optimization. TL, YW, and CZ contributed to the statistical analysis and drafting of the manuscript. ML, MM-B, and XH critically revised the manuscript. All authors read and approved the final manuscript.

## Conflict of Interest

The authors declare that the research was conducted in the absence of any commercial or financial relationships that could be construed as a potential conflict of interest.

## Publisher’s Note

All claims expressed in this article are solely those of the authors and do not necessarily represent those of their affiliated organizations, or those of the publisher, the editors and the reviewers. Any product that may be evaluated in this article, or claim that may be made by its manufacturer, is not guaranteed or endorsed by the publisher.
